# Optimized Ex Vivo Human Liver Slice Culture Maintains Extended Viability and Function for Hepatotoxicity Testing

**DOI:** 10.1002/advs.202523976

**Published:** 2026-06-29

**Authors:** Huiche Feng, Cinzia Esposito, Andrej Benjak, Matthias S. Matter, Miriam Cieri, Camilla De Carlo, Philipp Sedlaczek, Fabian Haak, Mattia Marinucci, Gabriel Fridolin Hess, Otto Kollmar, Mairene Coto‐Llerena, Luca Di Tommaso, Visar Vela, Lukas Bubendorf, Luigi M. Terracciano, Charlotte K. Y. Ng, Salvatore Piscuoglio

**Affiliations:** ^1^ Institute of Pathology University Hospital Basel Basel Switzerland; ^2^ Department for BioMedical Research (DBMR) University of Bern Bern Switzerland; ^3^ IRCCS Humanitas Research Hospital Rozzano Milan Italy; ^4^ Clarunis Department of Visceral Surgery University Center for Gastrointestinal and Liver Diseases St. Clara Hospital and University Hospital Basel Basel Switzerland; ^5^ Department of Visceral Transplant, Thoracic and Vascular Surgery University Hospital of Leipzig Leipzig Germany; ^6^ Visceral Surgery and Precision Medicine Research Laboratory Department of Biomedicine University of Basel Basel Switzerland; ^7^ Department of Biomedical Sciences Humanitas University Pieve Emanuele Milan Italy; ^8^ Centre Suisse de Contrôle de Qualité Geneva Switzerland

**Keywords:** drug‐induced liver injury, ex vivo liver model, extended tissue viability, hepatotoxicity testing, human precision‐cut liver slices

## Abstract

Human precision‐cut liver slices (hPCLS) are widely used to study liver physiology and disease, but their short lifespan limits long‐term functional studies and drug testing. Here, we describe an optimized ex vivo culture system, hPCLS‐EV (Extended Viability), that maintains tissue viability, cellular composition, and liver‐specific functions for up to five days. Optimization combined reduced slice thickness, high oxygen partial pressure, and air‐liquid interface culture, preserving hepatocyte integrity, stromal stability, and immune cell populations. Functional assays confirmed sustained albumin secretion, glutathione content, and cytochrome P450 activity. Transcriptomic profiling revealed rapid downregulation of metabolic pathways by day 3, followed by stabilization up to day 5, with limited advanced injury or fibrosis‐related remodeling. We applied hPCLS‐EV to model drug‐induced liver injury using acetaminophen and troglitazone. The model captured compound‐specific injury patterns, including early glutathione depletion with acetaminophen and delayed oxidative stress with troglitazone. Together, these findings support optimized hPCLS‐EV as a useful ex vivo human liver model that preserves key tissue architecture and selected liver functions over an extended culture period, enabling longitudinal assessment of hepatotoxic injury. This platform may provide a useful experimental tool for preclinical hepatotoxicity testing, mechanistic studies of liver injury, and patient‐specific applications in precision medicine.

## Graphical abstract

An optimized ex vivo human liver slice culture system preserves tissue architecture, hepatocyte function, and immune‐stromal complexity for at least five days. By refining slice thickness, oxygenation, air‐liquid interface, and extracellular matrix support, the platform enables human‐relevant hepatotoxicity testing and mechanistic studies, offering a complementary, human‐relevant model alongside animal and dissociated cell culture systems.

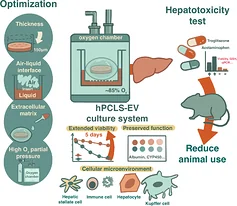

## 1 Introduction

The liver is a crucial organ responsible for vital functions such as protein synthesis, metabolism, and detoxification, making it a central focus of research in toxicology, pharmacology, and disease modelling [1]. For decades, preclinical drug screening studies have relied heavily on experimental animal models [2]. However, animal models are limited by ethical concerns, restricted tissue availability, high cost, and species‐specific differences [3]. On the other hand, traditional in vitro models like hepatocyte monolayers and immortalized cell lines are widely used, but they fail to reproduce the complex, multicellular architecture and physiological conditions of the human liver [4, 5, 6]. In particular, conventional hepatocyte monocultures do not adequately capture immune‐parenchymal interactions, and more complex co‐culture systems are often required to include inflammatory components in liver models [7, 8]. To overcome these limitations, ex vivo cultures of patient‐derived liver slices, known as human precision‐cut liver slices (hPCLS), have emerged as an organotypic human liver model. Compared to in vitro models, hPCLS maintains the native three‐dimensional tissue structure and multiple resident cell types, including hepatocytes, Kupffer cells, stellate cells, and immune populations [9, 10]. This organotypic model more closely recapitulates the natural human liver microenvironment and in vivo function and provides a human platform for preclinical high‐throughput drug testing [11, 12, 13]. Incorporating hPCLS into preclinical pipelines may, therefore, contribute to a reduction in animal use.

Despite the advantages, sustaining hPCLS tissue viability and functionality over time remains a major challenge. Current culture systems often suffer from rapid tissue necrosis, loss of cellular function, and disruption of the microenvironment, which limit their use in long‐term studies [14, 15]. Consistent with these observations, a comprehensive review reported that about 80% of hPCLS studies culture slices for ≤48 h [16]. Only a few studies achieve longer survival. Among these, Paish et al. used a simple perfused bioreactor to improve oxygenation and flow, extending the healthy lifespan of rat and human slices and enabling more reliable fibrosis modelling [17]. Importantly, while 24–48 h is sufficient for many acute toxicity readouts (e.g., oxidative stress, cytotoxicity, early metabolic suppression), extended culture is required for slower mechanisms and longitudinal functional endpoints to permit repeated dosing and time‐course analyses. Therefore, there is a need to optimize hPCLS culture conditions and establish practical workflows to maintain the integrity and functionality of liver slices over extended periods of time. This requires careful consideration of factors such as local equipment, oxygen delivery, culture methods, and tissue handling, all of which can influence culture performance across laboratories.

The liver is the primary site of drug metabolism and detoxification. Consequently, it is highly vulnerable to drug‐induced liver injury (DILI), particularly when exposed to high drug concentrations and reactive metabolites [18]. DILI can severely impair liver function and remains a leading cause of clinical trial failure, precautionary drug warnings, and post‐marketing withdrawals [19]. Supporting this, Onakpoya IJ, et al. documented 462 post‐marketing withdrawals worldwide between 1953 and 2013, with hepatotoxicity the most common reason [20]. Examples include nefazodone [21], bromfenac [22], and troglitazone [23], which were withdrawn in several countries for causing severe liver injury. Hepatotoxicity is therefore a major concern in drug development, and the early identification of drug candidates with high DILI risk is crucial [24, 25]. Developing more informative preclinical liver models that can support predictive assessment of hepatotoxicity is therefore important.

Here, we aimed to establish a practical and systematically optimized ex vivo culture workflow for human liver slices that improves tissue preservation, maintains key features of the liver microenvironment, and supports selected liver‐specific functions over an extended culture period. Rather than focusing on a single culture modification, we systematically evaluated slice thickness, oxygenation, air‐liquid interface culture, and extracellular matrix support to identify a baseline configuration that can be readily implemented in standard laboratory settings. We then characterized the model using complementary histological, functional, immunological, and transcriptomic readouts, including donor‐level analyses, to assess both tissue preservation and culture‐associated adaptation. Finally, we tested the applicability of the model for hepatotoxicity studies using acetaminophen and troglitazone. Together, these analyses establish a practical baseline for maintaining and evaluating human liver slices over five days, supporting their use in longitudinal studies of liver function, tissue adaptation, and hepatotoxic injury. By strengthening a human tissue‐based ex vivo approach, this work also contributes to broader efforts to reduce reliance on animal testing in preclinical liver research.

## 2 Materials and Methods

### 2.1 Tissue Samples

Human liver tissues were obtained from the non‐tumor resection margin of patients undergoing hepatectomy for various underlying diseases (Table 1 and Table S2). All procedures were conducted in accordance with the Declaration of Helsinki and approved by the local ethics committee (Ethics Committee of Basel, BASEC 2019–02118). Written informed consent was obtained from all patients. Human liver tissue collection, processing, and reporting follow the BRISQ (Biospecimen Reporting for Improved Study Quality) recommendations for key pre‐analytical variables. Immediately after resection, liver tissue was transferred to the pathology department of University Hospital Basel (Universitätsspital Basel, Basel, Switzerland) according to institutional routine. Once diagnostic sampling was completed, surplus non‐tumorous liver tissue was released for research use under prior consent. Tissue blocks of approximately 1–3 cm^3^ were immersed in ice‐cold slicing medium (Krebs–Henseleit buffer, 11 mm D‐glucose, 1.2 mm MgSO_4_, 1.2 mm KH_2_PO_4_, 4.7 mm KCl, 118 mm NaCl, 1.25 mm CaCl_2_, 25 mm NaHCO_3_; pH 7.4) and transported on ice to the slicing laboratory. The median time from resection to processing was 1.0 h (IQR 0.8–4.5). During transport and processing, specimens were kept at 4°C and protected from air exposure to limit warm ischemia. All sample processing was performed on‐site at University Hospital Basel. Donor‐level values are reported for the conditions and assays in which each donor was included, with detailed allocation provided in Table S2.

**TABLE 1:** Baseline Characteristics of the Study Cohort.

Baseline characteristics	Median(IQR) or n(%)
**Total number of donors**	**36**
Age (years)	66 (56–73)
Male sex	26 (72.2%)
**Surgery type**	
CRC liver metastasis	32 (88.9%)
Cholangiocarcinoma	4 (11.1%)
**Specimen logistics**	
Time from resection to processing (h)	1.0 (0.8–4.5)^a^
**Background liver histology**	
Mild/low‐grade steatosis noted	2 (5.6%)
Moderate/severe steatosis	0 (0%)
Advanced fibrosis/cirrhosis	0 (0%)
Viral hepatitis	0 (0%)

^a^ For two donors with processing time recorded as >5 h, a value of 5 h was used for descriptive summary statistics.

### 2.2 Tissue Slice Preparation

**FIGURE 1:**
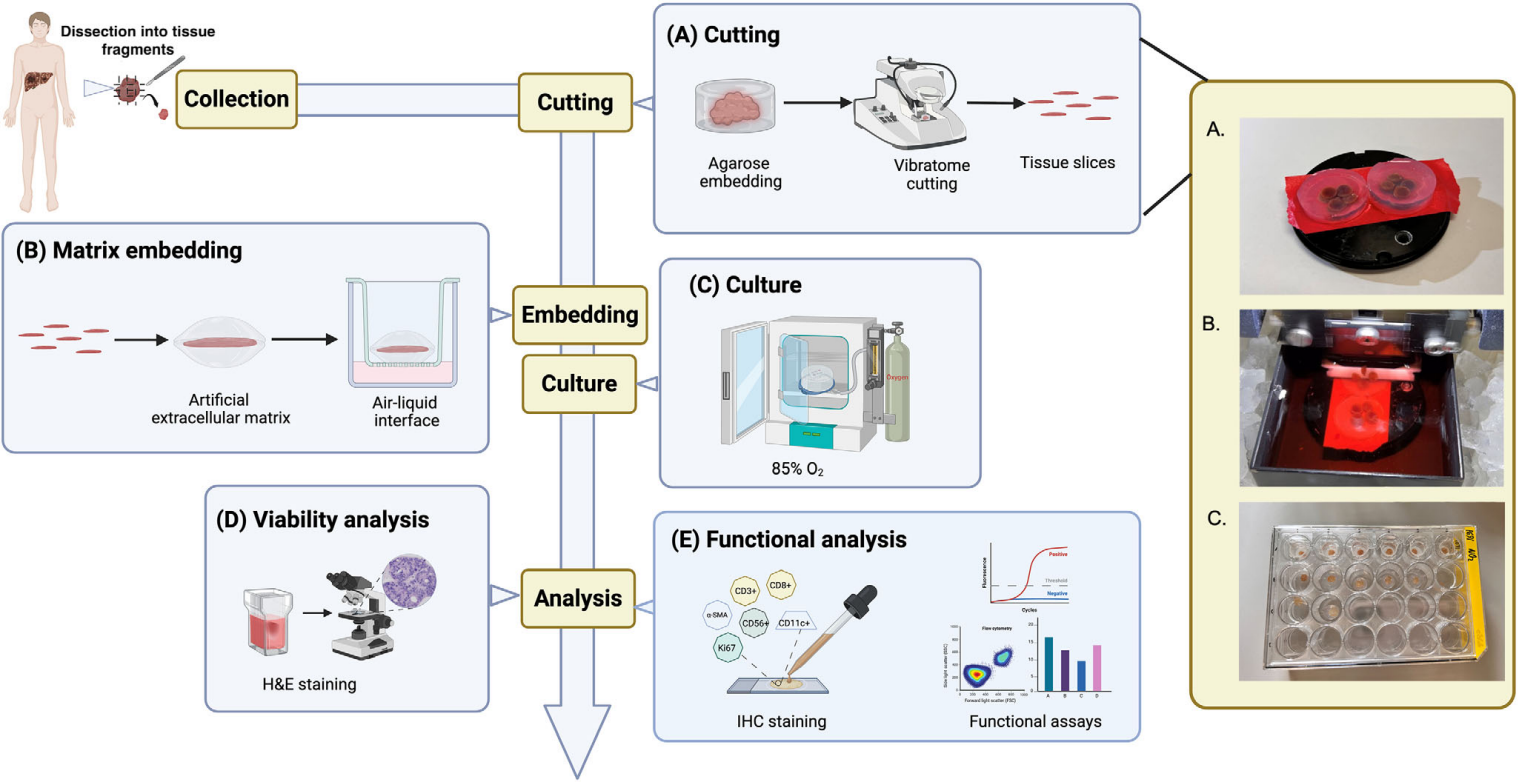
Workflow of the hPCLS‐EV System. To standardize sampling and culture, we implemented a five‐step workflow: collection of surgical liver tissue and dissection into fragments; vibratome cutting after agarose embedding; culture under defined oxygen and interface conditions; optional matrix embedding to stabilize slices; and downstream analyses for viability (H&E) and function (IHC, immune/microenvironment readouts, albumin and urea production, GSH content, CYP activity, and hepatotoxicity testing).

Human liver tissue was prepared as previously described [26]. Tissue cores were obtained using a 6‐mm Stiefel biopsy punch, transferred to a metal mold, embedded in 3% low‐gelling‐temperature agarose, and placed on ice for 2–5 min. Agarose‐embedded cores were sectioned using a Leica VT1200S vibrating blade vibratome at a speed of 0.3 mm/s, amplitude of 1.3 mm, and thickness ranging from 150 to 350 µm. Sections were cut in ice‐cold PBS supplemented with 10 µm ROCK inhibitor (Figure 1).

Human precision‐cut liver slices (hPCLS) were then embedded in an artificial extracellular matrix as previously described [27]. The matrix consisted of liver tissue culture medium supplemented with Cultrex Basement Membrane Extract (BME, 4 mg/mL; R&D Systems, Cat# 3433‐005‐02) and collagen type I (1 mg/mL; Corning, Cat# 354249). Slices were subsequently cultured in liver tissue culture medium composed of William's Medium E (WME) with L‐glutamine (Thermo Fisher Scientific, Cat# A1217601), supplemented with D‐glucose (14 mm; Sigma–Aldrich, Cat# G8769), penicillin (100 U/mL) and streptomycin (100 µg/mL; Gibco, Cat# 15140122), insulin (30 nm; Sigma–Aldrich, Cat# I9278), glucagon (100 nm; Sigma–Aldrich, Cat# G2044), corticosterone (1 µm; Sigma–Aldrich, Cat# C2505), epidermal growth factor (EGF, 1 nm; PeproTech, Cat# AF‐100‐15), and human AB serum (5%; Sigma–Aldrich, Cat# H4522). All experiments under controlled oxygen conditions were conducted in an O_2_/CO_2_ control chamber (ProOx Model C21; BioSpherix). Human precision‐cut liver slices were cultured ex vivo and evaluated longitudinally for up to 5 days, as shown in Figure 1 and Figure S1. Day 0 (D0) refers to the pre‐culture baseline measured after slicing and prior to incubation; later time points (e.g., day 3 or day 5) refer to the duration after culture initiation. For drug‐induced liver injury (DILI) experiments, hPCLS were first equilibrated for 1 h in drug‐free liver tissue culture medium and were then treated with troglitazone (10–50 µm; MedChemExpress, Cat# HY‐50935) or acetaminophen (APAP, 10–50 mm; MedChemExpress, Cat# HY‐66005) at the indicated concentrations. Stock solutions were prepared in DMSO and diluted in culture medium to the desired final concentrations (maximum DMSO ≤0.1% v/v); control slices received vehicle alone [28, 29]. Unless otherwise specified, treatments were initiated after the 1 h equilibration period and maintained for the indicated culture duration, with medium (and drug) refreshed every 24 h.

### 2.3 Hematoxylin & Eosin (H&E) Staining for Viability Assessment

Liver tissue morphology and viability were evaluated using hematoxylin and eosin (H&E) staining as described previously [30]. Human precision‐cut liver slices were collected at days 0, 3, and 5, fixed in 4% paraformaldehyde (PFA; Sigma‐Aldrich, USA) at 4°C for 24 h, and embedded in paraffin. Sections (4–6 µm) were deparaffinized, rehydrated, and stained with hematoxylin (8 min; Sigma–Aldrich, USA), followed by counterstaining with eosin (30 s; Sigma–Aldrich, USA). Slides were then dehydrated, cleared in xylene, and mounted with permanent mounting medium (Thermo Fisher Scientific, USA).

### 2.4 Viability Assessment and MTS Assay

Tissue viability was assessed based on histological criteria, including preservation of hepatocyte architecture, absence of widespread pyknotic nuclei, and minimal cytoplasmic vacuolization. This histology‐based approach allowed direct assessment of tissue architecture and spatial injury patterns and was therefore used as the primary viability readout. Morphology and viability were independently and blindly evaluated by four pathologists (MC, CDC, LDT and LMT), using a brightfield microscope (Leica DM2500, Germany). To complement the histological assessment, an MTS assay was performed using the MTS‐based colorimetric assay (CellTiter 96 AQueous One Solution, Promega, Figure S2A). MTS has been used as a viability‐related metabolic readout in tissue culture and precision‐cut tissue slice studies and was therefore included here as a complementary assay rather than a replacement for histological assessment [14, 16, 31]. Liver slices were incubated with the MTS reagent according to the manufacturer's instructions, and absorbance was read at 490 nm using a Varioskan LUX multimode microplate reader (Thermo Fisher Scientific). The background signal was corrected using reagent‐only blanks. For each donor, MTS values were normalized to the corresponding day 0 (D0) sample and expressed as a percentage of D0. Measurements were performed in technical duplicates

### 2.5 Measurement of Dissolved Oxygen in the Culture Medium

Dissolved oxygen in the culture medium was measured to characterize oxygen availability under the different culture conditions. Measurements were performed using a FireSting‐PRO oxygen meter (PyroScience, Germany), which recorded oxygen concentration (mg/L) together with sample temperature. Culture medium was equilibrated under the same conditions used for hPCLS culture, either in a standard incubator or in the oxygen chamber, and the oxygen probe was then immersed directly into the medium. Oxygen values were recorded continuously for approximately 1 min to allow stabilization of the signal, and the stabilized value was used for analysis. Absolute dissolved oxygen values are reported in mg/L.

### 2.6 Immunohistochemistry (IHC)

Paraffin‐embedded hPCLS were processed for immunohistochemistry (IHC) as described previously [32]. Sections were deparaffinized, rehydrated, and subjected to antigen retrieval using either citrate buffer (pH 6.0) or Tris‐EDTA buffer (pH 9.0) at 95°C for 15 min. After blocking with 5% bovine serum albumin (BSA) or 10% normal goat serum, sections were incubated overnight at 4°C with the primary antibodies listed in Table S1. The following day, sections were incubated with horseradish peroxidase (HRP)‐conjugated secondary antibodies for 1 h at room temperature, followed by development with 3,3′‐diaminobenzidine (DAB) substrate (Vector Laboratories, USA). Nuclei were counterstained with hematoxylin, and slides were dehydrated and mounted with permanent mounting medium. Slides were scanned using a Pannoramic MIDI II digital slide scanner (3DHISTECH) at 40× magnification, producing images with a resolution of 0.25 µm/pixel.

Cell quantification was performed by calculating the percentage of marker‐positive cells relative to the total number of hematoxylin‐stained nuclei in five randomly selected high‐power fields (HPFs, 40×) per section, in biological duplicates. Quantification was independently and blindly evaluated by two pathologists with the staining results (MC and CDC), and in cases of disagreement, a third pathologist performed a consensus review. ImageJ (NIH, USA) and QuPath (version 0.5.1) were used as supportive tools for image visualization and field assessment.

### 2.7 Liver Functional Assays

Functional assays were performed on subsets of donors based on tissue availability and assay format: albumin and urea were measured in donor‐matched culture supernatants from six donors (P19–P22, P33, P34); intracellular GSH, CYP3A4, and CYP2E1 were measured on tissue lysates from four donors (P19–P22); and CYP2C9 was measured on intact viable liver slices from four donors (P33–P36) using a separate reagent‐incubation protocol. Detailed donor allocation across assays is provided in Table S2.

#### 2.7.1 AST, ALT, Albumin, and Urea Assays

Aspartate aminotransferase (AST) and alanine aminotransferase (ALT) activities, as well as albumin secretion and urea production, were measured in culture supernatants. AST and ALT activities were quantified using the AST Assay Kit (MAK055, Sigma–Aldrich, USA) and ALT Assay Kit (MAK052, Sigma–Aldrich, USA), respectively. Albumin concentrations were determined using a human albumin enzyme‐linked immunosorbent assay (ELISA; RAB0603, Sigma–Aldrich, USA). Urea production was measured using the Urea Assay Kit (ab83362, Abcam, UK), according to the manufacturer's instructions.

#### 2.7.2 Glutathione (GSH) Levels

Intracellular glutathione (GSH) levels in hPCLS were measured using the GSH‐Glo^TM^ Glutathione Assay (Promega, Cat# V6911), according to the manufacturer's instructions for tissue extracts. Briefly, hPCLS were homogenized in ice‐cold PBS containing 2 mm EDTA, and the supernatant was collected after centrifugation. Aliquots (50 µL) were mixed with 50 µL of 2× GSH‐Glo^TM^ reagent and incubated at room temperature for 30 min. Subsequently, 100 µL of Luciferin Detection Reagent was added, and luminescence was measured after 15 min using a Varioskan LUX multimode plate reader (Thermo Fisher Scientific). GSH concentrations were calculated by interpolation from a glutathione standard curve.

#### 2.7.3 Protein Quantification

Total protein concentration in tissue lysates was determined using the Pierce BCA Protein Assay Kit (Thermo Fisher Scientific, Cat# 23227) according to the manufacturer's instructions and was used for normalization of CYP activity data.

#### 2.7.4 Cytochrome P450 3A4 (CYP3A4) Activity

CYP3A4 enzymatic activity was measured using the CYP3A4 Activity Assay Kit (Fluorometric; Abcam, Cat.# ab211076), according to the manufacturer's protocol for tissue lysates. Briefly, hPCLS were homogenized in CYP3A4 assay buffer, centrifuged at 15,000 × g for 15 min at 4°C, and the supernatant was collected. For each sample, 50 µL of lysate was incubated with an NADPH‐generating system and CYP3A4 substrate mix in a white 96‐well plate. Fluorescence (Ex/Em = 535/587 nm) was recorded for 30–45 min at 37°C using a Varioskan LUX multimode plate reader (Thermo Fisher Scientific). Resorufin formation was quantified using a resorufin standard curve, and CYP3A4 activity was corrected for incubation time and normalized to total protein content determined by BCA assay, with final values expressed as pmol product/min/mg of protein.

#### 2.7.5 Cytochrome P450 2E1 (CYP2E1)‐Associated Activity

CYP2E1‐associated activity was assessed using a fluorometric coumarin‐based assay adapted from previously described methods [33]. Briefly, tissue lysates were incubated with 7‐MFC in the presence of NADPH, and the formation of the fluorescent product was quantified using a 7‐hydroxy‐4‐(trifluoromethyl)coumarin (HFC) standard curve. Sodium diethyldithiocarbamate trihydrate (DDC; Sigma‐Aldrich, Cat# 106689) was used in selected experiments as an inhibitor control. Enzymatic activity was corrected for incubation time and normalized to total protein content determined by BCA assay, with final values expressed as pmol product/min/mg protein.

#### 2.7.6 Cytochrome P450 2C9 (CYP2C9) Activity

CYP2C9‐associated activity was assessed using the P450‐Glo^TM^ CYP2C9 Assay System (Promega, Cat# V8791) with minor adaptations for intact live hPCLS. Briefly, individual live hPCLS were transferred to a white 96‐well plate and incubated with CYP2C9 assay reagent at a volume adjusted to tissue wet weight (equivalent to 100 µL reagent per 20 mg wet tissue) at 37°C for 4 h. Following incubation, 50 µL of the reaction mixture was transferred to a fresh white 96‐well plate, mixed with Luciferin Detection Reagent according to the manufacturer's instructions, and luminescence was measured using a Varioskan LUX multimode plate reader (Thermo Fisher Scientific). Because this assay provides a luminescence‐based relative readout rather than absolute product quantification, CYP2C9 results were used for comparative analysis across conditions and are presented as blank‐corrected relative luminescence units (RLU).

### 2.8 RNA Extraction and Quantitative Real‐Time PCR

Four slices per condition were collected for RNA extraction; RNA and DNA were extracted from the tissue slices using the Quick‐DNA/RNA Miniprep Plus Kit (Zymo, cat#D7003), following the manufacturer's instructions [34]. The RNA purity and concentration were measured using NanoDrop spectrophotometer (Thermo Fisher Scientific, USA), and RNA integrity was verified by agarose gel electrophoresis.

Complementary DNA (cDNA) was synthesized from 500 ng of total RNA in a final volume of 20 µL using the SuperScript^TM^ IV VILO Master Mix (Thermo Fisher Scientific, Cat# 11766050, USA). Reverse transcription was performed under the following conditions: 25°C for 10 min, 50°C for 10 min, and enzyme inactivation at 85°C for 5 min.

Quantitative Real‐Time PCR (qPCR) was performed on a QuantStudio 6 Flex Real‐Time PCR System (Applied Biosystems, Thermo Fisher Scientific, USA) using PowerUp SYBR Green Master Mix (Thermo Fisher Scientific, USA). Each reaction contained 2 µL of cDNA, 0.5 µm forward and reverse primers, and 10 µL of SYBR Green Master Mix, in a total volume of 20 µL. The thermal cycling protocol consisted of an initial denaturation at 95°C for 10 min, followed by 40 cycles of denaturation at 95°C for 15 s, annealing at 60°C for 30 s, and extension at 72°C for 30 s.

Primers were designed using Primer‐BLAST (NCBI). The primer sequences used in this study are listed in Table S3.

Gene expression levels were quantified using the comparative cycle threshold (Ct) method (ΔΔCt method) as previously described [35]. Ct values for each target gene were normalized to the housekeeping gene GAPDH using the following calculations:

$$\Delta Ct=Ct_{target}\;-Ct_{GAPDH}$$

$$\Delta \Delta Ct=\Delta Ct_{sample}-\Delta Ct_{control}$$

$$Relative\;expression=2^{-▵▵Ct}$$

### 2.9 Data and Statistical Analysis

The experimental design included donor‐matched hPCLS cultures across conditions and time points. Unless otherwise specified, data are presented as mean ± standard deviation (SD), and n indicates the number of biological replicates, defined as independent donors. For each donor, replicate slices or assay measurements from the same condition and time point were averaged before statistical analysis, and statistical tests were performed on donor‐level values.

For two‐condition donor‐matched comparisons, data were analyzed using paired tests. For comparisons across multiple culture conditions or time points, repeated‐measures or non‐parametric matched analyses were used as appropriate, with the specific test for each dataset reported in the corresponding figure legend. Specifically, marker‐positive cell populations across D0, D3 and D5 were analyzed using the Friedman test with Dunn's multiple‐comparison correction. Functional readouts comparing control and optimized hPCLS‐EV conditions were analyzed on D3 and D5 values using two‐way repeated‐measures ANOVA with Geisser–Greenhouse correction, followed by Šídák's multiple‐comparisons test. D0 was shown as the matched uncultured baseline and was not included in the multiple comparisons. Adjusted *p* values are reported where multiple‐comparison correction was applied.

Normality was assessed where appropriate. For normally distributed paired data, a two‐tailed paired t‐test was used; for non‐normally distributed paired data, the Wilcoxon signed‐rank test was used. For unpaired comparisons, Welch's t‐test was used where appropriate. A *p* value < 0.05 was considered statistically significant. All statistical analyses were performed using GraphPad Prism v9.4.1 (GraphPad Software, San Diego, CA, USA).

### 2.10 RNA Sequencing and Analysis

RNA sequencing was performed by BGI (Shenzhen, China) using the DNBSEQ Eukaryotic Strand‐Specific Transcriptome Resequencing Kit on the DNBSEQ platform, generating 150 bp paired‐end reads. For each sample, a median of 33.5 million read pairs was obtained (range: 28.3–36.2 million). Raw sequencing reads were aligned to the human reference genome (hg38, annotated with gencode.v33.primary_assembly.annotation.gtf) using STAR (version 2.7.3a) [36]. The resulting BAM files were processed using RSEM (version 1.3.2) for gene‐level quantification [37]. Differential gene expression analysis was performed in R (version 4.2.2) using the DESeq2 package (version 1.38.3), based on RSEM‐derived count data [38]. Principal component analysis (PCA) and heatmap visualization were conducted using variance‐stabilizing transformed counts. Gene set enrichment analysis (GSEA) was performed using the fgsea package (version 1.24.0) [39].

## 3 Results

### 3.1 Optimization of Ex Vivo Culture Conditions Extends Liver Slice Viability

Maintaining viable and functional ex vivo liver tissue is challenging because of limited oxygen and nutrient diffusion, progressive cellular stress, and loss of tissue integrity. Conventional hPCLS models typically lose viability within two days (48 h), restricting their use in long‐term experiments [16, 40]. To address this, we systematically optimized culture parameters to establish a standardized hPCLS workflow that enhances survival and function (hPCLS‐EV), focusing on oxygenation, nutrient diffusion, and tissue stability (Figure 1).

We enrolled 36 adult liver donors undergoing surgical resection for colorectal liver metastases (88.9%) or cholangiocarcinoma (11.1%) (Table 1 and Table S2). Non‐tumorous liver parenchyma was sampled from macroscopically normal areas at least 10 mm from the lesion margin, avoiding major vessels and areas of necrosis. For each case, a board‐certified pathologist reviewed hematoxylin‐eosin (H&E) sections from the resection specimen to confirm non‐tumorous background liver histology. To minimize any confounding effects, we prospectively excluded donors with chronic liver disease (cirrhosis; viral, autoimmune, or cholestatic hepatitis); moderate/severe steatosis; active systemic inflammation/sepsis; recent liver‐directed therapy or chemotherapy within a predefined washout; prolonged cold ischemia beyond our threshold; or inadequate specimen mass/quality. We retained two cases with mild steatosis, as it is common in surgical livers and is not expected to affect short‐term slice viability or hepatocyte function.

To isolate effects of individual culture parameters, we used a single‐variable design: each test altered one parameter while keeping a fixed baseline (250 µm slices, atmospheric oxygen ∼21% O_2_, matrix embedding). H&E‐based viability scoring was used as the primary readout for the optimization experiments. This was supported by a matched pilot comparison with MTS metabolic viability readouts, which showed a strong positive association across matched slice samples (Spearman ρ = 0.93, p = 0.00024; Figure S2A).

**FIGURE 2:**
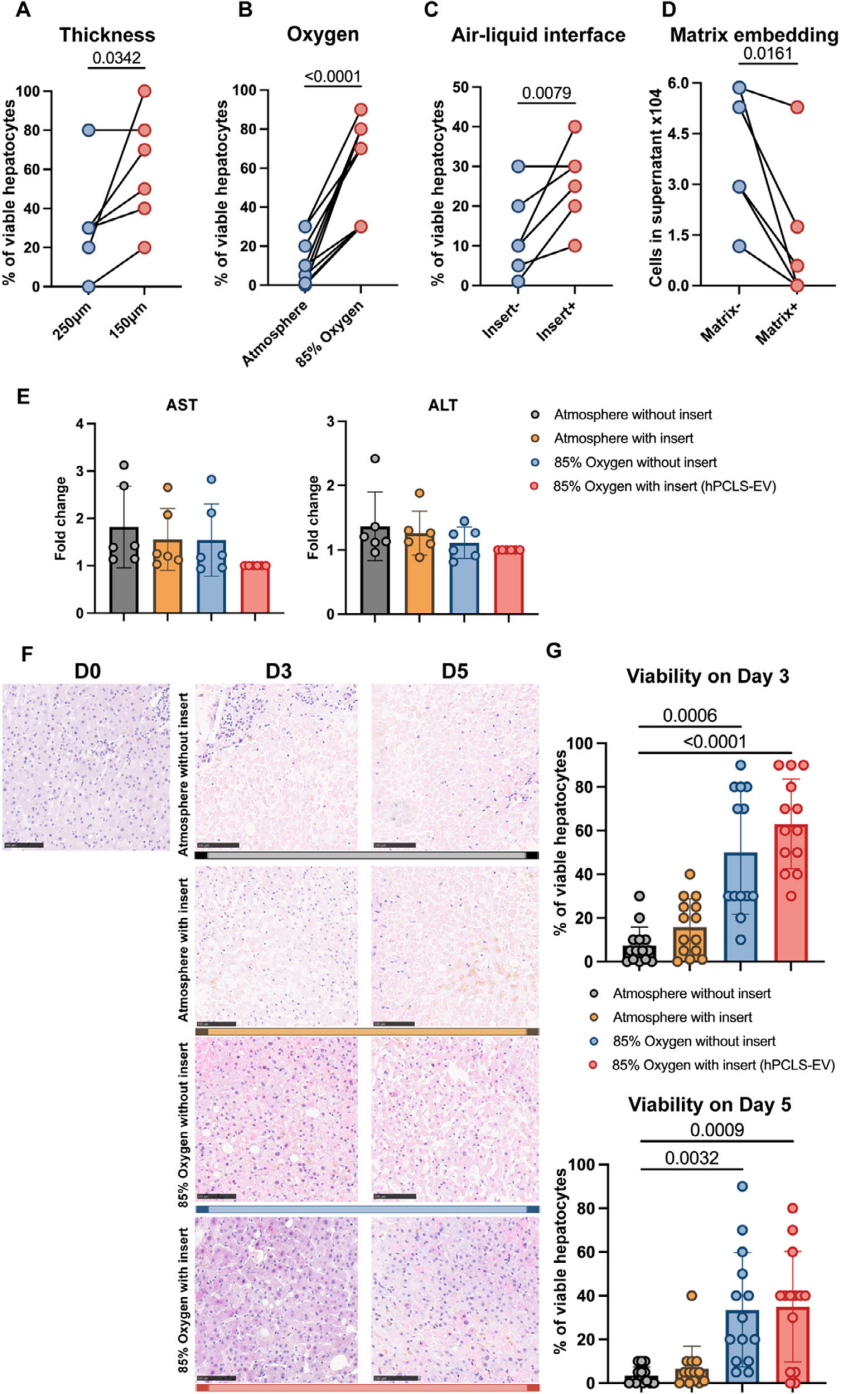
Optimization of ex vivo liver slice culture conditions. Viability was assessed by H&E staining after three days of culture under different conditions: (A) slice thickness comparison between 150 and 250 µm, *n* = 8 independent donors; (B) high oxygen partial pressure, 85% O_2_ vs atmospheric, *n* = 11 independent donors; (C) air‐liquid interface using porous inserts, n = 8 independent donors; and (D) matrix embedding assessed by cell efflux into the medium, *n* = 6 independent donors. For A–D, each dot represents the mean value from one donor, derived from three donor‐matched slices per condition where available. Differences were analyzed using paired tests; *p* values are indicated. Parameters were subsequently combined into an optimized culture protocol. (E) ALT and AST activity measured at 24 h in different conditions, normalized to the optimized group, *n* = 6 independent donors. Bars show mean fold change ± s.d. Each dot represents the mean value from one donor, derived from donor‐matched slice supernatants per condition. (F) Representative H&E staining of slices cultured for 3 and 5 days under the different conditions. Scale bars, 100 µm. Time points: D0 represents the pre‐culture baseline; D3–D5 represent post‐initiation culture time points. (G) Quantification of hepatocyte viability on day 3 and day 5, *n* = 14 independent donors. Bars show mean values ± s.d. Dots represent donor‐level means derived from three donor‐matched slices per condition where available. Statistical analysis was performed on donor‐level means using repeated‐measures one‐way ANOVA with donor as the matching factor for each time point, followed by correction for multiple comparisons. P values are shown.

We first examined the effect of tissue thickness, as this directly influences nutrient and oxygen penetration. Excessive thickness can lead to central necrosis, whereas overly thin slices increase the proportion of damaged cells [26, 41]. The standard thickness of 250 µm corresponds to approximately 15 hepatocyte layers [16]. Reducing slice thickness to 150 µm (∼10 hepatocyte layers) improved viability after three days of culture, as demonstrated by hematoxylin and eosin (H&E) staining, likely due to enhanced oxygen and nutrient accessibility (Figure 2A).

Given the high oxygen demand of hepatocytes due to their central role in xenobiotic metabolism, we next tested the impact of oxygen concentration. Morphologically, slices cultured in 85% oxygen retained tissue structure with fewer necrotic areas compared with those maintained under atmospheric oxygen (∼21%), which showed progressive cellular disintegration (Figure 2B,F). These results indicate that elevated oxygen partial pressure improves survival by reducing hypoxia‐induced damage. To better define oxygen availability under our culture conditions, we measured absolute dissolved oxygen levels in the culture medium. Medium maintained in the oxygen chamber (85%) reached dissolved oxygen levels of approximately 18–22 mg/L, whereas medium kept in a standard incubator with atmospheric oxygen (21%) remained at approximately 5–8 mg/L (Figure S2B). To further facilitate oxygenation, we cultured slices on porous inserts to establish an air‐liquid interface. Compared with fully submerged slices, the air‐liquid interface condition preserved tissue structure more effectively and reduced necrosis, suggesting that direct oxygen exposure enhances viability over extended culture periods (Figure 2C,F).

We also evaluated the effect of matrix embedding. Embedding slices in an artificial extracellular matrix decreased the number of cells released into the medium, indicating reduced leakage and improved tissue stability (Figure 2D), consistent with previous studies using Matrigel hydrogel [27, 42].

Finally, we combined the most effective parameters, reduced slice thickness (150 µm), elevated oxygen (85%), and culture at an air‐liquid interface with matrix embedded as shown in the workflow (Figure 1) to evaluate their collective impact on tissue survival and liver function. AST and ALT release after 24 h was lower under optimized conditions, although the difference was not statistically significant (Figure 2E). By histology, however, slices cultured under the optimized protocol showed a clear survival advantage: hepatocyte viability averaged 60% at day 3 compared with ∼10% in controls (250 µm, submerged, atmospheric oxygen), and 40% at day 5 compared with complete degradation in controls (0%) (Figure 2F,G). Interestingly, at atmospheric oxygen level, the air‐liquid interface significantly improved viability, whereas at 85% oxygen rate this effect was attenuated, suggesting that increased oxygen supply can compensate for the absence of an air‐liquid interface. Additionally, we also examined slice wet weight and protein content across time points and culture conditions (Figure S2C,D). Wet tissue weight ranged from 1.8 to 5.5 mg per slice, with group means between 3.41 and 4.12 mg across D0, D3 and D5. Protein content was broadly comparable across groups, with median values ranging from 10.20 to 16.15 µg/mg tissue. No consistent time‐dependent shift or systematic difference between control and optimized hPCLS‐EV conditions was observed, supporting comparable tissue input for downstream functional assays.

Together, these findings demonstrate that coordinated optimization of culture parameters substantially extends hPCLS viability, providing a practical ex vivo culture configuration suitable for longer‐term studies. Besides, this optimized configuration requires no specialized perfusion equipment and can be implemented using standard laboratory incubators (Figure S1). This distinguishes the system from bioreactor‐based approaches, offering a simpler and more scalable solution for most research environments.

### 3.2 Preservation of Hepatocyte, Stromal, Endothelial, and Immune Cell Populations

**FIGURE 3:**
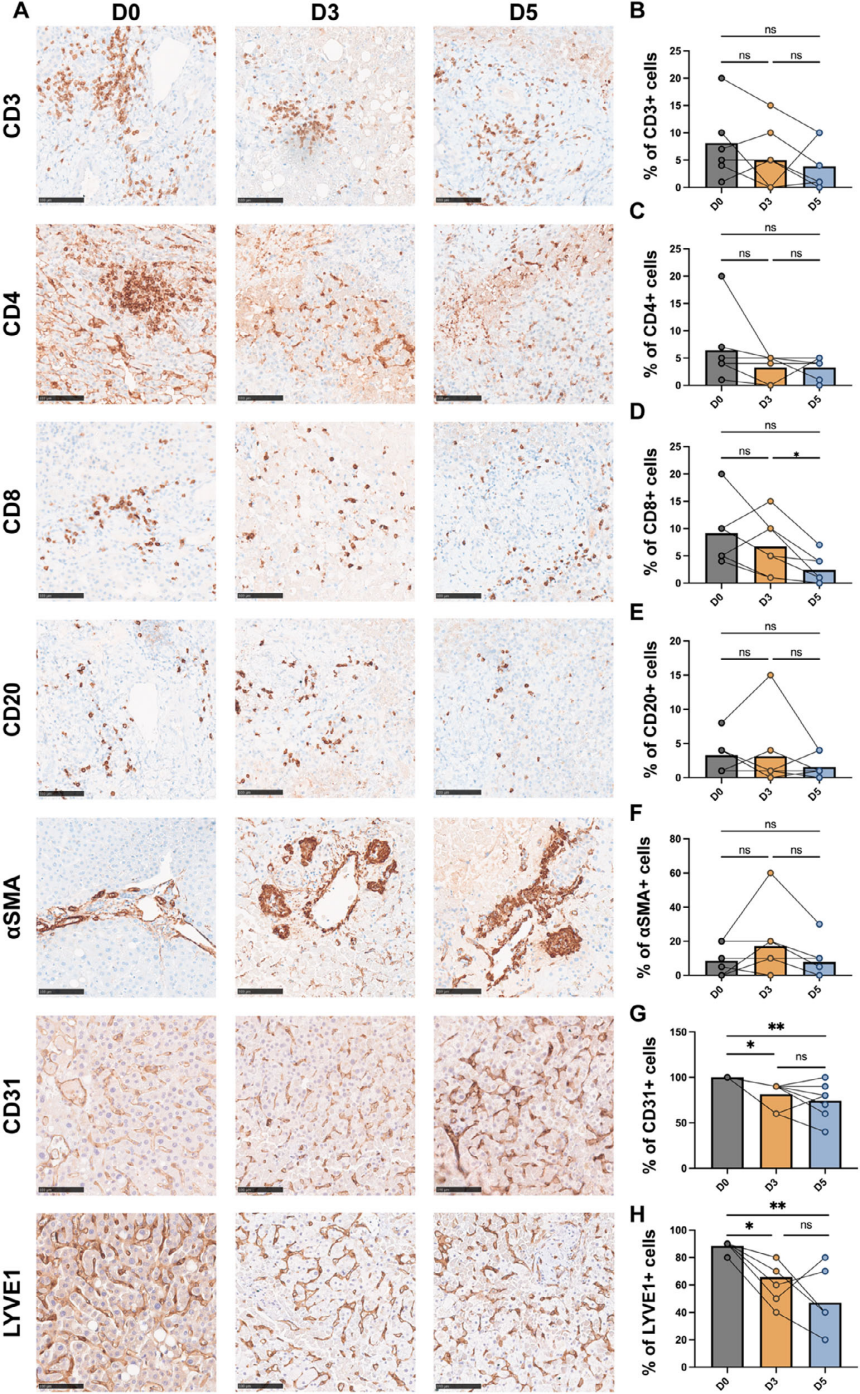
Preservation of cellular microenvironment complexity under optimized culture conditions. Liver tissue slices from seven independent donors were analyzed at D0, D3, and D5 by immunohistochemical staining for CD3, CD4, CD8, CD20, αSMA, CD31, and LYVE1. D0 represents the pre‐culture baseline; D3 and D5 represent post‐initiation culture time points. (A) Representative images of stained sections at each time point. Scale bars, 100 µm. (B–H) Quantitative analysis of marker‐positive cell populations across culture time points, *n* = 7 independent donors. Each dot represents the mean value from one donor, derived from two donor‐matched slices where available. Bars show mean percentages. Statistical analysis was performed on donor‐level values using the Friedman test with Dunn's multiple‐comparison correction. ns = not significant; ^*^ *p* < 0.05, ^**^ *p* < 0.01.

To determine whether the optimized culture conditions maintained not only hepatocyte viability but also the complexity of cellular composition of the liver microenvironment, we performed immunohistochemical (IHC) staining at day 0, day 3, and day 5 in samples from seven donors. A panel of lineage‐specific markers was selected to capture both parenchymal and non‐parenchymal cell types. HepPar‐1 was used to identify hepatocytes, while αSMA marked activated stellate cells and myofibroblasts. Endothelial cells were assessed using CD31, while LYVE1 was included as a more specific marker of liver sinusoidal endothelial cells (LSECs). The immune compartment was assessed using CD3 (total T cells), CD4 (helper T cells), CD8 (cytotoxic T cells), CD56 (natural killer cells), CD68 (Kupffer cells/monocyte–macrophages), CD20 (B cells), and CD11c (hepatic dendritic cells). Ki‐67 was included as a proliferation marker to assess whether culture induced cell expansion (Figure 3 and Figure S3).

Quantitative analysis showed that HepPar‐1‐positive hepatocyte regions remained readily detectable throughout culture, indicating that the parenchymal compartment was still represented within the retained tissue architecture (Figure S3A,B). Ki‐67 positivity remained consistently low across all time points (Figure S3A,C), demonstrating that the culture system preserved the largely non‐proliferative state of mature liver tissue in vivo and did not drive culture‐induced proliferation. Similarly, αSMA^+^ stromal cells showed no significant increase, suggesting that culture did not trigger spontaneous activation of fibrogenic pathways (Figure 3A,F). Similarly, the immune landscape was also broadly preserved in hPCLS‐EV model (Figure S3G). The densities of CD3^+^ T cells, including CD4^+^ and CD8^+^ subsets, as well as CD56^+^ natural killer cells, CD68^+^ Kupffer cells, CD20^+^ B cells, and CD11c^+^ dendritic/myeloid cells, showed no significant changes over time (Figure 3 and Figure S3). This indicates that immune cell populations, which are often poorly preserved in conventional culture systems, were stably maintained in our optimized model. Finally, CD31^+^ endothelial staining remained broadly detectable throughout the culture period, with only a modest reduction over time (∼74% of baseline remaining at day 5; Figure 3A,G). LYVE1^+^ LSECs were also retained at days 3 and 5, despite some reduction from baseline (∼47% of baseline remaining at day 5; Figure 3A,H).

Taken together, these findings show that the optimized hPCLS‐EV culture system preserves not only hepatocyte integrity but also the complexity of the native microenvironment, including stromal cells, resident immune populations, and endothelial compartments. This provides a relevant platform for applications requiring preserved cell‐cell interactions, such as studies of immunotoxicity, liver disease mechanisms, and DILI.

### 3.3 Preservation of Liver‐Specific Functions Under Optimized Culture Conditions

Hepatocytes account for approximately 80% of total liver mass and are responsible for a wide range of essential functions, including glycogen storage, cholesterol synthesis and transport, urea metabolism, detoxification of xenobiotics, and the production of lipids and serum proteins [43]. To evaluate whether hepatocyte‐specific functions were preserved during culture, we measured albumin secretion, an indicator of hepatic functionality [44, 45], and urea production, an indicator of hepatocyte ureogenic capacity and ammonia detoxification function [46], in supernatants collected at different time points.

**FIGURE 4:**
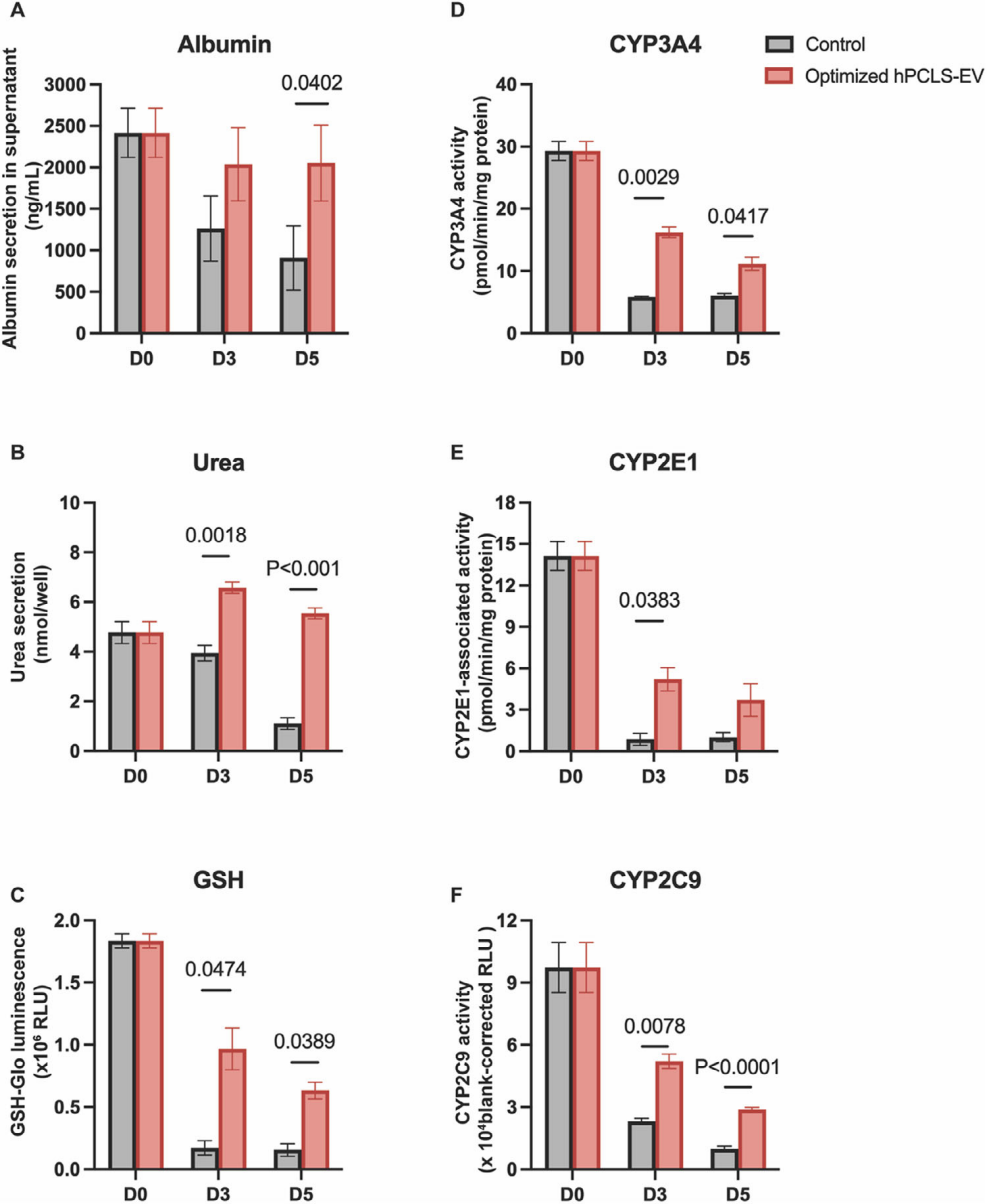
Preservation of tissue functionality under optimized culture conditions. Functional stability of liver tissue slices was assessed at D0, D3, and D5 under control and optimized hPCLS‐EV conditions. D0 represents the pre‐culture baseline; D3 and D5 represent post‐initiation culture time points. (A) Albumin secretion in culture supernatants, n = 6 independent donors (P19–P22, P33, P34). (B) Urea production in culture supernatants, *n* = 6 independent donors (P19–P22, P33, P34). (C) Intracellular glutathione (GSH) levels, *n* = 4 independent donors (P19–P22). (D) CYP3A4 activity, *n* = 4 independent donors (P19–P22). (E) CYP2E1‐associated activity, *n* = 4 independent donors (P19–P22). (F) CYP2C9 activity, *n* = 4 independent donors (P33–P36). Data are shown as mean ± s.d. of donor‐level values. For each donor, replicate slices or assay measurements from the same condition and time point were averaged before statistical analysis. Statistical comparisons between control and optimized hPCLS‐EV conditions were performed on D3 and D5 values using a two‐way repeated‐measures ANOVA with Geisser–Greenhouse correction, followed by Šídák's multiple‐comparisons test. Adjusted *p* values are shown.

As expected, albumin secretion declined over time, consistent with the progressive loss of functional activity inherent to ex vivo culture (Figure 4A). However, this decline was substantially attenuated under the optimized conditions compared with conventional culture. Albumin levels in conventional cultures had already decreased by day 3 and were markedly reduced by day 5, whereas slices maintained in the optimized system showed better preservation of albumin output over the same period. A similar pattern was observed for urea production, where urea levels were comparable between groups at day 0 but were significantly higher in the optimized condition at days 3 and 5, indicating better preservation of hepatocyte metabolic function over time (Figure 4B). We further measured intracellular glutathione (GSH) levels, an important antioxidant and marker of detoxification, which were also better preserved under optimized culture conditions (Figure 4C).

To determine whether hepatic drug metabolism was maintained during ex vivo culture, we measured CYP3A4‐ and CYP2E1‐associated activity in hPCLS at day 0 and after culture under optimized or control conditions (Figure 4D,E). Although enzyme activity decreased over time, as commonly observed after tissue isolation and in vitro culture [47], both CYP3A4‐ and CYP2E1‐associated activities were consistently better preserved in slices cultured under optimized conditions than in control slices. In addition, CYP2C9 activity, measured on intact viable liver slices from a separate donor cohort (P33‐P36) because the liver slice format required incubation of reagents with viable tissue, showed the same general directional pattern as the lysate‐based CYP3A4 and CYP2E1 assays, with a higher CYP2C9‐associated luminescence signal under optimized conditions than under control conditions (Figure 4F).

These findings indicate that while some decline in liver‐specific functional activity occurs over time in ex vivo systems, optimized culture conditions substantially improve the preservation of hepatocyte synthetic and metabolic functions. The better maintenance of albumin secretion, urea production, key CYP activities, and GSH content supports the value of the optimized culture conditions for sustaining hepatic function and cellular homeostasis over extended culture. Together, these results further support the value of the optimized hPCLS‐EV model for drug toxicity studies and disease modeling.

### 3.4 Transcriptomic Adaptation of hPCLS‐EV During Ex Vivo Culture

**FIGURE 5:**
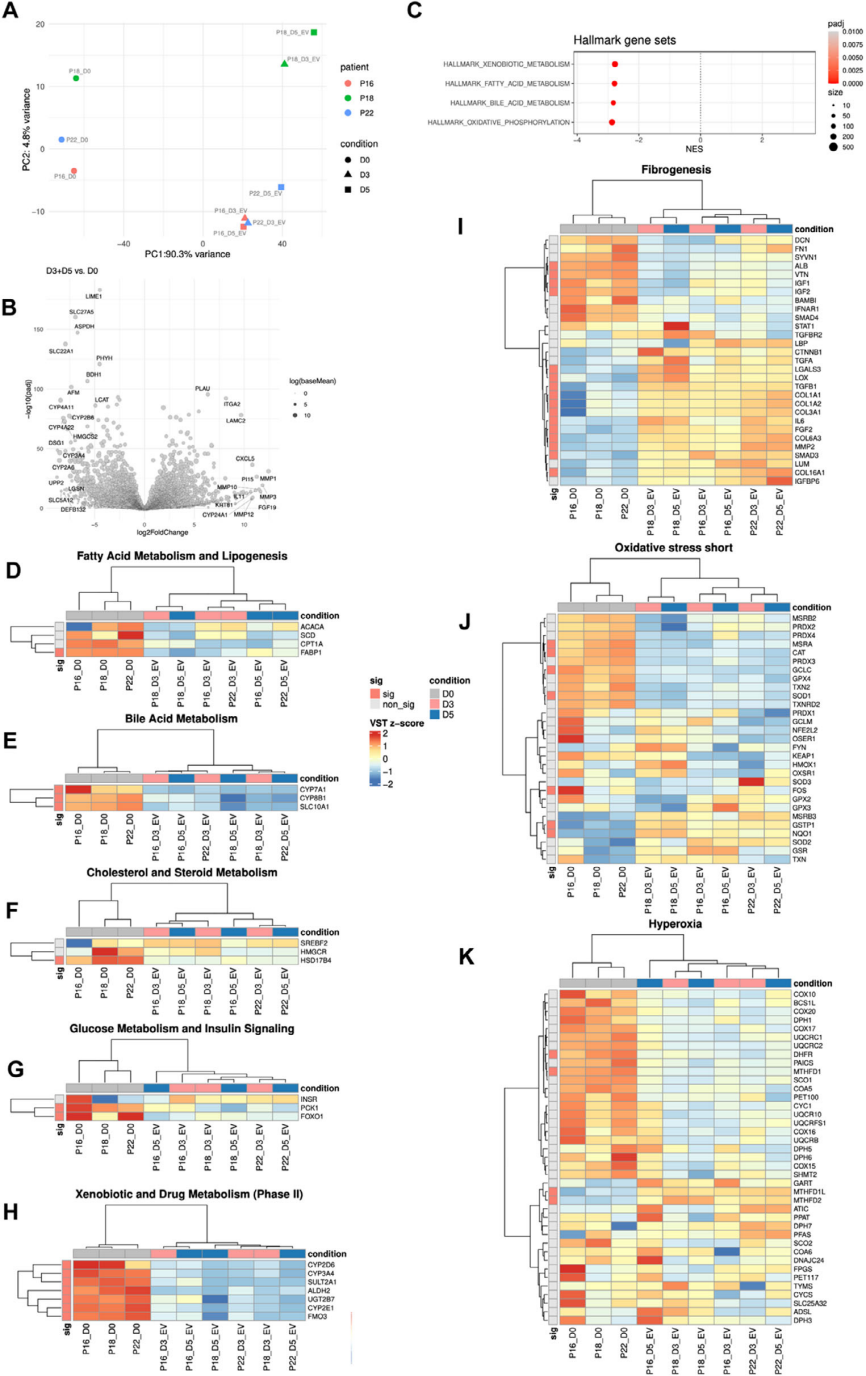
Transcriptomic characterization of ex vivo cultured liver tissue under optimized conditions. RNA sequencing was performed on matched liver tissue slices from three independent donors (P16, P18, and P22) at D0, D3 and D5. D0 represents the pre‐culture baseline; D3 and D5 represent post‐initiation culture time points. (A) Principal component analysis of donor‐level transcriptomic profiles. Samples are colored by donor and shaped by time point; each symbol represents one RNA‐seq sample from one donor at one time point. (B) Volcano plot of differentially expressed genes (DEGs) comparing (D3 + D5) with D0. The top 20 significantly upregulated and downregulated DEGs are indicated, based on log_2_ fold change. (C) Gene set enrichment analysis (GSEA) of significantly enriched biological pathways. Heatmaps illustrate expression changes in selected genes involved in: (D) fatty acid metabolism and lipogenesis, (E) bile acid metabolism, (F) cholesterol and steroid metabolism, (G) glucose metabolism and insulin signaling, (H) xenobiotic and drug metabolism, (I) fibrogenesis, (J) oxidative stress, and (K) hyperoxia‐related genes. Columns represent donor‐level RNA‐seq samples from P16, P18, and P22 at each time point, and expression is shown as variance‐stabilized z‐scores.

To characterize transcriptional dynamics during ex vivo culture, we performed RNA sequencing of hPCLS‐EV at days 0, 3, and 5 in three donors. Principal component analysis (PCA) revealed clear separation of samples by culture duration (Figure 5A). Day 0 samples clustered distinctly from day 3 to day 5, indicating major transcriptional shifts immediately after the transition to ex vivo conditions. Days 3 and 5 samples clustered more closely together, suggesting that most changes occurred within the first three days and stabilized thereafter. Donor‐specific clustering was also observed, highlighting inter‐individual variability as an important determinant of transcriptional profiles.

Differential expression analysis comparing days 3/5 to day 0 identified thousands of significantly regulated genes (log_2_FC ≥ 1.5, padj ≤ 0.01; Figure 5B and Table S4), consistent with a previous report of extensive culture‐associated transcriptomic remodeling in hPCLS [15]. Inflammation‐ and extracellular matrix (ECM)‐related genes, including *CXCL5*, *MMP1*, *MMP3*, *MMP10*, and *MMP12*, were strongly upregulated, reflecting tissue stress and early wound‐healing responses. In contrast, key metabolic genes, particularly those encoding cytochrome P450 enzymes (e.g., *CYP2A6*, *CYP4A11*, *CYP3A4*, *CYP4A22*), were markedly downregulated, indicating a decline in metabolic capacity. Gene set enrichment analysis (GSEA) further confirmed broad suppression of metabolic pathways, including fatty acid metabolism, bile acid metabolism, and xenobiotic clearance (Figure 5C).

Closer inspection of genes in selected metabolic pathways showed differential sensitivity to the ex vivo environment. Genes involved in fatty acid metabolism and lipogenesis, such as *ACACA*, *SCD*, and *CPT1A*, remained relatively stable, whereas *FABP1* was strongly downregulated (Figure 5D). In bile acid metabolism, *CYP7A1*, *CYP8B1*, and *SLC10A1* were sharply suppressed by day 3, with minimal further reduction thereafter (Figure 5E). Cholesterol and steroid metabolism exhibited mixed regulation: *HSD17B4* was strongly downregulated, while regulatory genes such as *SREBF2* and *HMGCR* remained largely unchanged (Figure 5F). Glucose metabolism and insulin signaling showed similar selectivity: gluconeogenic genes (*PCK1*, *FOXO1*) declined significantly, whereas *INSR* remained stable (Figure 5G).

Drug metabolism networks were particularly affected. By day 5, Phase I enzymes (*CYP3A4*, *CYP2E1*, *CYP2D6*) and Phase II enzymes (*FMO3*, *UGT2B7*, *SULT2A1*, *ALDH2*) all showed consistent downregulation (Figure 5H). Despite this, functional assays demonstrated that CYP450 activity was maintained under optimized culture conditions (Figure 4D–F), suggesting that post‐transcriptional regulation, residual protein stores, or low degradation rates may sustain enzymatic activity. This discrepancy highlights the importance of integrating transcriptomic data with functional readouts when assessing the reliability of the model.

Since hPCLS are widely used to study fibrosis [16], we also examined the expression of fibrogenesis‐related genes (Figure 5I). Collagen transcripts (*COL1A1*, *COL1A2*, *COL3A1*, *COL6A3*, *COL16A1*) were significantly upregulated, consistent with ECM remodeling, whereas fibronectin‐related genes (*FN1*, *DCN*, *LUM*) remained unchanged. Similarly, TGF‐related genes (*BAMBI*, *SMAD4*, *TGFA*, *TGFBR2*) showed only limited modulation, indicating partial but not full activation of fibrogenic pathways. These findings align with morphological results showing limited collagen accumulation without overt fibrotic remodeling within five days of culture (Figure 2F and Figure S3A).

Finally, we examined whether prolonged culture under elevated oxygen was associated with a distinct oxidative stress or hyperoxia‐related transcriptional response. Transcriptomic profiling did not identify a dominant pattern consistent with clear hyperoxia‐driven injury under the optimized condition (Figure 5J,K). Classical antioxidant and redox genes, such as *CAT, PRDX2, PRDX3, PRDX4, GCLC, GPX4, TXN2, SOD1*, and *TXNRD2*, did not show a consistent increase during culture. Similarly, NRF2‐associated genes, including *NFE2L2, KEAP1, HMOX1, NQO1*, and *GCLM*, varied across donors and time points. Among the hyperoxia‐related genes, several mitochondrial genes, including *COX10, COX17, UQCRC1, UQCRC2, UQCRFS1*, and *SCO1*, were generally higher at day 0, whereas others such as *MTHFD1L, MTHFD2, ATIC, PFAS*, and *TYMS* changed without a clear common trend across the samples. Overall, these results do not point to a strong signal of oxygen‐driven injury under the optimized condition. Instead, the changes were more consistent with general ex vivo adaptation of the tissue, together with persistent donor‐specific differences.

In summary, our transcriptomic analysis shows that ex vivo culture induces an early wave of transcriptional reprogramming characterized by downregulation of metabolic pathways and activation of wound‐healing responses. These changes stabilize after three days, and under optimized conditions, functional metabolic capacity is preserved despite reduced expression of distinct metabolic genes. Limited activation of fibrosis‐ and injury‐related pathways further supports the suitability of this culture system for maintaining liver tissue integrity during extended culture.

### 3.5 Modelling Drug‐Induced Liver Injury (DILI) in Optimized hPCLS‐EV

To test whether the optimized ex vivo culture system can support drug toxicity testing, we exposed liver slices to well‐characterized hepatotoxicants. Acetaminophen (APAP) and Troglitazone (TGZ) were selected as reference compounds since they represent commonly used hepatotoxicants with distinct and well‐described liver injury profiles in in vitro hepatotoxicity studies [48, 49]. As a proof‐of‐principle for tissue‐level hepatotoxicity studies, we aimed to assess whether the optimized hPCLS‐EV system can detect distinct compound‐associated injury responses under controlled conditions.

**FIGURE 6:**
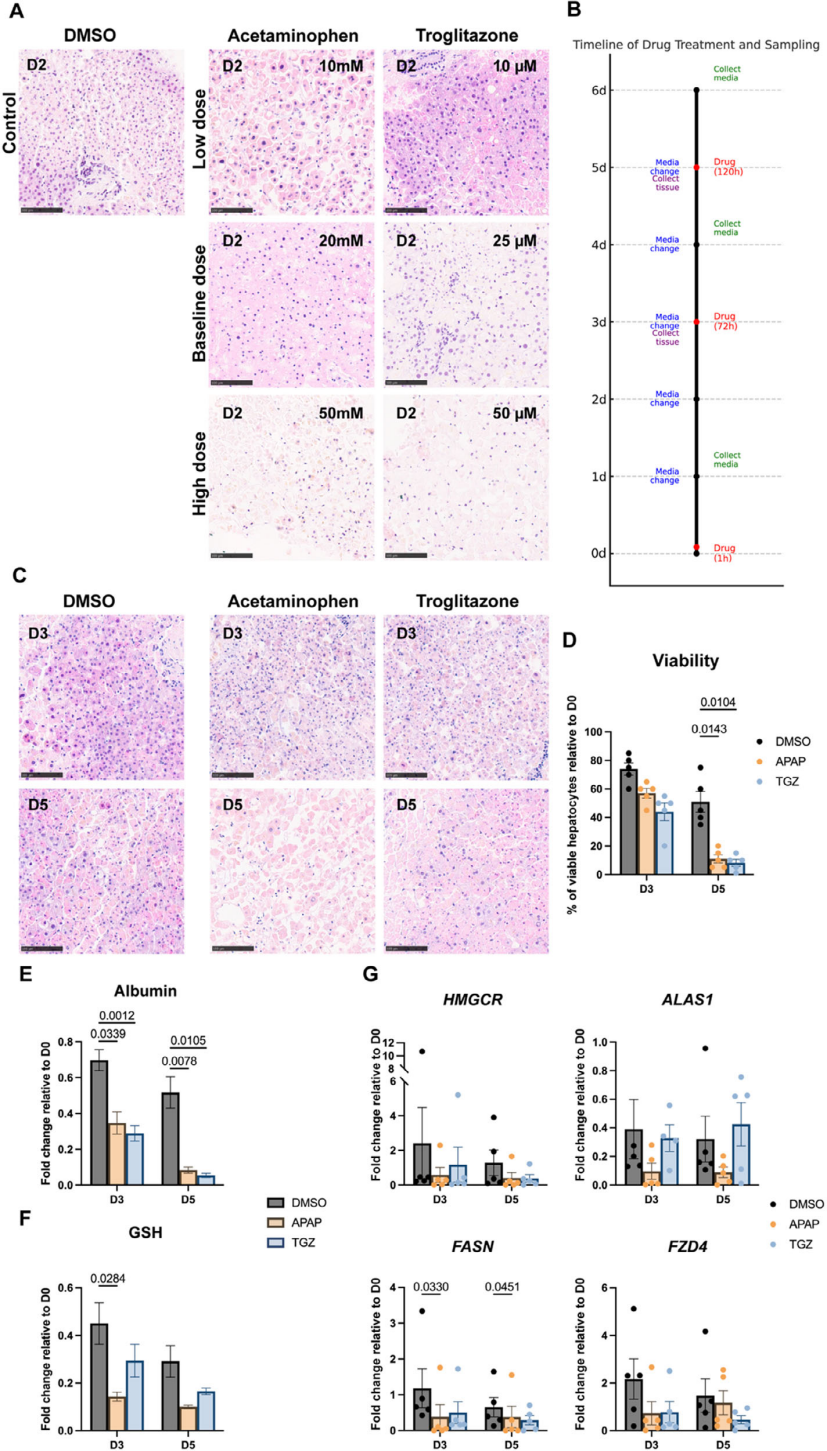
Drug‐induced liver injury modeling in optimized ex vivo cultured liver slices. Liver slices from five independent donors were cultured under optimized hPCLS‐EV conditions and treated with DMSO, acetaminophen (APAP) or troglitazone (TGZ). D0 represents the matched pre‐treatment baseline used for normalization; D3 and D5 represent post‐initiation culture time points. (A) Representative H&E images showing tissue morphology after two‐days exposure to different doses of APAP (10, 20, 50 mm) and TGZ (10, 25, 50 µm), compared with untreated controls. Scale bars, 100 µm. The 20 mm APAP and 25 µm TGZ conditions were selected for subsequent experiments. (B) Experimental timeline of drug treatment, medium changes and sample collection. (C) Representative H&E images of APAP‐ and TGZ‐treated slices compared with DMSO controls at D3 and D5. Scale bars, 100 µm. (D) Quantification of viable hepatocytes, (E) albumin production, (F) glutathione (GSH) content, and (G) qPCR of representative metabolic/stress‐related genes (*HMGCR*, *ALAS1*, *FASN*, *FZD4*) on Day 3 and Day 5, expressed as fold change relative to Day 0, showing drug‐specific transcriptional responses. Data are shown as mean ± s.d. Each dot represents one donor‐level value. For each donor, replicate slices or assay measurements from the same treatment and time point were averaged before statistical analysis. Statistical comparisons were performed at each time point on donor‐level values using one‐way ANOVA with Dunnett's multiple‐comparisons test. Adjusted *p* values are shown.

We first conducted two‐day dose–response experiments with APAP (10, 20, 50 mm) and TGZ (10, 25, 50 µm). Histological analysis by H&E staining revealed that ≥20 mm APAP and ≥25 µm TGZ induced evident morphological alterations, including nuclear condensation and hepatocellular damage (Figure 6A). These thresholds are consistent with concentrations used in other PCLS and hepatocyte‐based DILI studies (APAP [48, 50]; TGZ [51, 52]). Based on these results, 20 mm APAP and 25 µm TGZ were selected for follow‐up experiments.

Guided by previous time‐course experiments in primary mouse hepatocytes [53], we analyzed early (day 3) and late (day 5) responses in slices from five independent donors (Figure 6B). On day 3, overall hepatocyte viability was not significantly reduced (Figure 6C,D). However, in contrast to DMSO‐treated slices, early morphological changes, including nuclear condensation and tissue disintegration, were already apparent in APAP‐ and TGZ‐treated slices (Figure 6C). By day 5, both compounds induced a dramatic reduction in viable hepatocyte numbers.

Functional assays corroborated these findings. Albumin secretion, a marker of hepatocyte synthetic capacity, decreased progressively in both treatment groups, with stronger suppression observed under APAP, consistent with more severe hepatocyte impairment (Figure 6E). On the other hand, GSH depletion occurred rapidly in APAP‐treated slices, reflecting the conversion of APAP to the reactive metabolite NAPQI (Figure 6F). In contrast, TGZ caused a delayed GSH decrease, becoming apparent on day 5, consistent with its distinct mechanism of hepatotoxicity through mitochondrial dysfunction and progressive oxidative stress.

We also examined the expression of stress‐ and metabolism‐related genes previously implicated in DILI. *HMGCR* and *FASN* transcripts, encoding key regulators of cholesterol and lipid biosynthesis, were downregulated after both APAP and TGZ, consistent with early suppression of metabolic programs under toxic stress [54, 55]. *FZD4*, a Frizzled receptor mediating Wnt/β‐catenin signals involved in hepatic injury‐repair and regeneration, was also reduced in both treatment groups [56, 57]. In contrast, *ALAS1*, the rate‐limiting enzyme in heme biosynthesis linked to CYP‐dependent metabolism [58], showed a downward trend in slices treated with APAP but not TGZ (Figure 6G). These gene expression patterns mirror known compound‐specific mechanisms: APAP‐induced suppression of heme biosynthesis via NAPQI toxicity, and TGZ‐mediated effects on lipid metabolism and mitochondrial function [55, 59, 60, 61].

Together, these data show that, under the optimized hPCLS‐EV conditions, distinct compound‐associated injury responses can be detected and maintained over five days, enabling extended longitudinal drug testing. In addition, these proof‐of‐principle results support the further investigation of hPCLS‐EV as a complementary tissue‐level mechanistic model for hepatotoxicity studies, while broader compound panels will be needed to establish its predictive performance for diverse DILI phenotypes.

## 4 Discussion

The use of precision‐cut liver slices has been well established since the reference protocol of de Graaf et al. in 2010 [26], and applications have since expanded to modeling diverse liver diseases [62, 63, 64, 65]. Although PCLS has been widely used in liver research, most hPCLS studies remain limited to short culture periods of approximately 24–48 h [16]. The current study describes the systematic optimization of a patient‐derived hPCLS culture workflow, resulting in improved tissue preservation, extended culture duration, and the maintenance of selected liver‐specific functions and microenvironmental features over five days. A key contribution is the transcriptomic characterization of hPCLS during prolonged culture, which revealed early culture‐associated adaptation and helped interpret functional preservation in the context of molecular changes over time. Finally, proof‐of‐principle experiments with acetaminophen (APAP) and troglitazone (TGZ) showed that distinct compound‐associated injury responses can be detected under the optimized culture conditions, supporting the further investigation of hPCLS‐EV as a complementary preclinical tool for mechanistic hepatotoxicity studies.

Many studies rely mainly on surrogate viability assays, such as LDH, ATP, MTS, or live/dead staining, which may not fully reflect hepatocyte‐specific function or overall tissue quality. In our study, we chose to use H&E as the primary viability readout, which was highly concordant with MTS readouts, because it allows direct evaluation of hepatocyte integrity, tissue architecture, and spatial injury patterns, while we added albumin secretion, urea production, GSH and CYP activity to provide functional readouts.

The substantial variability in culture conditions for hPCLS across laboratories, especially in terms of slice thickness, oxygenation, and culture setup, likely contributes to the variable viability and functional readouts reported across studies. Our systematic evaluation revealed that thinner slices, higher oxygen exposure, and air‐liquid interface culture each improved tissue integrity and reduced central necrosis, supporting oxygen availability and delivery as a major determinant in this system. However, oxygen conditions in PCLS culture are difficult to compare across studies because the gas‐phase oxygen setting does not directly reflect the oxygen environment experienced by the tissue [66]. In our system, the increase in liquid‐phase oxygen was markedly smaller than the difference suggested by gas‐phase settings. Moreover, oxygen exposure in PCLS is further shaped by tissue architecture and diffusion constraints, and modeling studies suggest that the internal oxygen gradient within a slice depends strongly on slice diameter, slice position in the well, and external oxygen conditions [41]. Thus, oxygen availability should be interpreted as a tissue‐level culture variable rather than directly compared with conditions in monolayer cell culture. Notably, because oxygen tension may influence injury mechanisms involving mitochondrial stress or oxidative damage, its potential impact on hepatotoxicity readouts should be considered when interpreting compound‐specific responses. For instance, hyperoxic conditions have been reported to accelerate APAP‐induced injury progression in primary mouse hepatocytes, while the underlying molecular mechanism appears to remain preserved [67]. Although this finding cannot be directly extrapolated to intact human liver slices, it remains a relevant cautionary example when comparing hPCLS‐derived injury kinetics with monolayer hepatocyte models.

One advantage of the hPCLS‐EV model is that it retains the native liver cellular context better than hepatocyte monocultures. This is important because liver responses to injury and drugs are shaped not only by hepatocytes but also by the microenvironment. In our workflow, matrix support was therefore used not only to stabilize the slices, but also to help preserve this multicellular composition during prolonged culture. Immune cell populations were largely stable over five days, whereas endothelial‐associated markers changed more over time, as LSECs are particularly difficult to maintain outside their sinusoidal niche. Previous studies have shown that LSEC marker expression can decline within 42–72 h, and that monolayer‐cultured LSECs can lose their phenotype within three days, whereas 3D or layered culture systems help delay this loss [68, 69, 70]. In our system, CD31 and LYVE1 remained detectable at day 5, indicating partial retention of endothelial compartments, although the LYVE1 decline shows LSEC features are still affected during culture. This work provides a useful basis for future studies of drug responses that involve inflammatory signaling, immune‐parenchymal interactions, or other non‐parenchymal cell contributions.

The functional assays further showed that the hPCLS‐EV model better preserved several complementary hepatocyte functions over time. The decline in albumin but relatively well‐maintained urea production suggests that hepatocyte synthetic output [44] and ammonia detoxification and nitrogen metabolism [71] may not deteriorate in parallel during ex vivo adaptation. GSH content, a readout for intracellular antioxidant reserve and redox status, also showed a decline over time, consistent with our transcriptomic data, in which classical antioxidant and NRF2‐associated genes (including *GCLC*, *GCLM*, *NFE2L2*) did not show a consistent upregulation during culture, suggesting that the antioxidant machinery is not sufficiently activated to compensate for ongoing oxidative or biosynthetic demands. In the context of APAP toxicity, GSH is of particular relevance as APAP bioactivation consumes GSH during detoxification of the reactive metabolite NAPQI [72], but the decline during ex vivo adaptation should be considered when interpreting the dynamic range of APAP‐induced GSH depletion in the toxicity experiments.

The CYP enzyme activity analyses also suggested a better preservation of drug‐metabolizing function under optimized conditions. In particular, CYP3A4 (CYP‐mediated xenobiotic metabolism [73]), CYP2E1 (APAP bioactivation and glutathione‐linked oxidative stress [74]) and CYP2C9 (a major clinically relevant drug‐metabolizing CYP [75]) consistently support maintenance of drug metabolic function in hPCLS‐EV. Notably, CYP2C9 was assessed in intact liver slices, allowing enzyme activity to be measured in viable tissue while preserving the native tissue context, in contrast to the lysate‐based CYP3A4 and CYP2E1 measurements. However, these metabolic readouts also require careful technical interpretation, as CYP activity may also be influenced by experimental components such as vehicle conditions, since solvents such as DMSO [76] or ethanol [77] can affect CYP activity even at low concentrations. In our DILI experiments, DMSO was used at ≤0.1% v/v and was matched across vehicle and treatment groups. Nevertheless, as DMSO can affect CYP activity and has been reported to inhibit CYP‐dependent APAP bioactivation [76, 78], solvent conditions should be considered when comparing drug sensitivity across different liver models. Future studies could consider DMSO‐free or lower‐DMSO solvent conditions for CYP‐dependent compounds where technically feasible. Moreover, CYP activity readouts should be normalized by tissue wet weight or total protein content rather than per slice, as the wet weight of individual slices can vary substantially (∼2 to ∼6 mg per slice in our study).

Our transcriptomic analysis demonstrates a sharp transcriptional rewiring within 3 days, followed by partial stabilization, suggesting an acute adaptation phase with injury and wound‐healing programs. The upregulation of fibrogenic and inflammatory gene sets may reflect culture‐induced injury, including unavoidable pre‐analytical factors such as cold ischemia, mechanical slicing, and the surgical background of donor tissues. However, collagen deposition and TGF‐β signaling remained limited, and H&E morphology together with α‐SMA staining did not show marked fibrotic remodeling, indicating that the model captures early injury responses without progressing to advanced fibrosis within five days. While metabolic genes, including cytochrome P450 enzymes and gluconeogenic regulators, were downregulated, selected functional readouts, including albumin secretion, urea production, GSH content, and CYP enzymatic activity, remained detectable. This difference between transcriptomic and functional readouts further supports the need for combined structural, molecular, and functional assessment when evaluating hPCLS quality. Importantly, we did not observe a global transcriptional pattern consistent with clear oxygen‐driven injury under the optimized condition. Instead, the changes in oxidative stress, oxygen adaptation, and inflammatory genes were mixed and are more consistent with broader ex vivo culture adaptation, although subtle or cell type‐specific oxygen‐related effects can still be present. Finally, the donor‐specific clustering indicates that hPCLS‐EV remains strongly shaped by interindividual variability, which should be considered when interpreting gene expression patterns and functional responses. At the same time, the donor‐specific patterns highlight how hPCLS‐EVs may be useful to model patient‐specific drug responses.

The hepatotoxicity assays provided proof‐of‐principle that optimized hPCLS‐EV can model distinct forms of DILI at the tissue level. APAP and TGZ were selected as representative hepatotoxicants with different mechanisms: APAP causes glutathione depletion following CYP‐mediated bioactivation to NAPQI, whereas TGZ is associated with mitochondrial dysfunction and disruption of lipid metabolic homeostasis. Indeed, we observed that APAP caused rapid GSH depletion and a downward trend of heme biosynthesis (*ALAS1*), consistent with metabolism to the toxic intermediate NAPQI, while TGZ induced delayed GSH depletion, mitochondrial dysfunction, and suppression of lipid metabolism genes *(HMGCR*, *FASN*). For the drug concentration, we selected 20 mM APAP and 25 µm TGZ by pilot titration to induce clear and reproducible tissue injury, since nominal concentrations cannot be directly compared across liver model systems [79, 80]. This is particularly important for hPCLS, in which compounds must distribute through an intact tissue environment with preserved multicellular structure and cellular barriers, rather than directly accessing isolated cells [16, 79, 81]. Thus, effective exposure and apparent drug sensitivity can be shaped by tissue penetration, intra‐slice distribution, culture setup, and donor‐ or slice‐level variability, which should be kept in mind when screening compounds of unknown toxicity. A similar principle has been reported in complex human liver spheroid models, where the presence of non‐parenchymal cells reduced APAP sensitivity compared with hepatocyte‐only spheroids, further supporting that multicellular context can influence dose‐related responses [82]. Clinical and cell‐based concentration ranges therefore provide useful context, but they cannot be directly equated with nominal concentrations applied to intact hPCLS. For APAP, overdose plasma concentrations are typically lower (∼1–4 mm peak) [83, 84], whereas toxicity in directly exposed hepatocyte‐based systems is commonly observed in the millimolar range, including 5–20 mM in primary human hepatocytes and TC_50_ values of approximately 5.8–6.1 mm after 24 h exposure in 3D HepaRG spheroids [48, 85] In contrast, TGZ toxicity is typically observed in the low‐to‐mid micromolar range, with toxic effects reported at 10–50 µm in human hepatocytes [86] and a TC_50_ of 46.2 µm after repeated exposure in 3D HepaRG spheroids [85]. Even in tissue‐based liver models, the nominal concentrations vary across studies and are often model‐ and endpoint‐dependent. For example, rodent PCLS study reported extensive viability loss at 10–15 mm APAP [81], whereas Kitamura et al. observed no detectable toxicity in normal rat liver slices exposed to up to 10 mm APAP for 24 h, and Vatakuti et al. selected 2.5 mm APAP as a toxic condition based on marked ATP‐based viability loss after 24 h exposure [87, 88]. For TGZ, comparable micromolar concentrations have been used in PCLS‐based inflammatory‐stress DILI models, including 15 µm in hPCLS and 30 µm in mPCLS [89]. Accordingly, nominal concentrations used in hPCLS should be interpreted in a model‐specific context, and the time required to observe a comparable toxic response may also differ between systems [79]. Together, these findings demonstrate that hPCLS‐EV can capture compound‐specific injury signatures associated with distinct DILI mechanisms, supporting its further development as a complementary tissue‐level model for mechanistic hepatotoxicity studies.

Our optimized ex vivo hPCLS‐EV platform can be applied to small surgical or core‐biopsy samples while preserving patient‐specific tissue context. By capturing morphological, functional, and transcriptomic readouts, the system captures compound‐specific injury patterns and may complement existing preclinical models for mechanistic hepatotoxicity studies and personalised liver research.

Despite these advances, several limitations remain. Although the optimized conditions extend the usable functional window of hPCLS‐EV, they did not fully prevent culture‐associated decline. Further refinement of medium composition, solvent conditions, or perfusion‐based culture may help maintain tissue stability and liver‐specific function for longer periods. Our functional characterization of albumin, urea, GSH, and multiple CYP‐related readouts still did not capture all aspects of hepatocyte biology. Further analysis of phase II metabolism, transporter activity, bile acid handling, and broader CYP profiling using MS‐based multiplex approaches would be of interest. In addition, although hPCLS‐EV preserved immune cell populations during culture, we did not directly assess immune cell activation or immune‐mediated drug responses. This will be important for future studies of compounds whose toxicity involves inflammatory signaling or immune‐mediated liver injury. Furthermore, this study provides proof‐of‐principle evaluating only two reference compounds (APAP and TGZ) rather than a comprehensive assessment of DILI. A broader compound panel covering additional injury mechanisms (e.g., cholestasis, idiosyncratic hepatotoxicity, and mitochondrial toxicity from other classes) will be needed to establish the predictive value of hPCLS‐EV for diverse DILI phenotypes.

In conclusion, this study presents an optimized ex vivo hPCLS‐EV culture workflow that supports the longitudinal assessment of human liver tissue and captures distinct hepatotoxic injury responses to APAP and TGZ. These findings support further evaluation of hPCLS‐EV as a complementary tissue‐level model for preclinical liver research.

## Author Contributions

S.P. and C.K.Y.N. conceived and supervised the study. H.F. designed and performed the ex vivo liver slice culture experiments, functional assays, and drug‐induced liver injury experiments, and analyzed the data. C.E., M.C.L. and M.M. contributed to the experimental work and methodology. A.B. and C.K.Y.N. performed the transcriptomic and bioinformatic analyses. M.S.M., M.C., C.D.C., L.D.T., V.V., L.B., and L.M.T. performed the histopathological and immunohistochemical evaluation. P.S., F.H., G.F.H., and O.K. provided surgical liver specimens and associated clinical information. H.F. and S.P. wrote the manuscript. All authors reviewed and approved the final version of the manuscript.

## Funding

L.D.T. is supported by AIRC grant number IG 2020 ID.25087. L.M.T. was supported by AIRC grant number IG 2019 ID.23615 and the “National Centre for HPC, Big Data and Quantum Computing” (CN00000013 ‐ Spoke 8), financed by NextGenerationEU PNRR MUR – M4C2 – Action 1.4 – Call “Potenziamento strutture di ricerca e di campioni nazionali di R&S”. C.K.Y.N. is supported by the AIRC Start‐Up grant number 30787. The funders had no role in study design, data collection and analysis, decision to publish, or preparation of the manuscript.

## Conflicts of Interest

M.S.M. has consulted for ThermoFisher, Merck, GSK, Janssen‐Cilag, Roche and Novartis; received speaker honoraria from Incyte Biosciences, Merini Group and Astellas; and has non‐commercial research agreements with AstraZeneca. L.B. has consulted for Amgen, Bayer, ThermoFisher, Janssen‐Cilag and AstraZeneca; received speaker honoraria from ThermoFisher, Janssen and Takeda; and has research agreements with Roche and Novartis.

## Supporting information




**Supporting File 1**: advs76268‐sup‐0001‐SuppMat.docx.

**Supporting File 2**: advs76268‐sup‐0002‐TableS2.xlsx.

**Supporting File 3**: advs76268‐sup‐0003‐TableS4.xlsx.

## Data Availability

The data that support the findings of this study are available within the article and its Supporting Information. Additional underlying numerical data (e.g., raw assay readouts exported from the plate reader as Excel files) are available from the corresponding author upon reasonable request.
