# Supplementary material for: Optimized Ex Vivo Human Liver Slice Culture Maintains Extended Viability and Function for Hepatotoxicity Testing
Source: Adv Sci (Weinh). 2026 Jun 29;13(52):e23976. doi: 10.1002/advs.202523976 (PMC13336509; doi:10.1002/advs.202523976)
Supplement: Supplementary file 1 [file ADVS-13-e23976-s001.docx]

**Supporting Information**

**Optimised Ex Vivo Human Liver Slice Culture Maintains Extended Viability and Function for Hepatotoxicity Testing**

*Huiche Feng^1^, Cinzia Esposito^1^, Andrej Benjak^2^, Matthias S Matter^1^, Miriam Cieri^3^, Camilla De Carlo^3^, Philipp Sedlaczek^4^, Fabian Haak^4,5^, Mattia Marinucci^1^, Gabriel Fridolin Hess^4^, Otto Kollmar^4^, Mairene Coto-Llerena^6^, Luca Di Tommaso^3,7^, Visar Vela^8^, Lukas Bubendorf^1^, Luigi M Terracciano^3,7^, Charlotte K Y Ng^3,7*^, Salvatore Piscuoglio^3,7*^*

# **Supplementary Tables**

| Marker | Cell Type | Supplier & Cat# | Dilution / Clone |
| --- | --- | --- | --- |
| Ki67 | Proliferation marker | Abcam ab16667 | 1:200 / Rabbit mAb (SP6) |
| CD3 | T cells | Abcam ab135372 | 1:200 / Rabbit mAb (SP162) |
| CD4 | Helper T cells | Abcam ab133616 | 1:200 / Rabbit mAb (EPR6855) |
| CD8 | Cytotoxic T cells | Abcam ab17147 | 1:200 / Mouse mAb (C8/144B) |
| CD11c | Dendritic cells | Abcam ab52632 | 1:500 / Rabbit mAb (EP1347Y) |
| CD68 | Macrophages | Abcam ab213363 | 1:200/ Rabbit mAb (EPR20545) |
| α‑SMA | Stellate cells | Sigma-Aldrich A2547 | 1:400 / Mouse mAb (1A4) |
| CD20 | B cells | Abcam ab78237 | 1:200 / Rabbit mAb (EP459Y) |
| CD56 | NK cells | Abcam ab75813 | 1:200 / Rabbit mAb (EP2567Y) |
| CD31 | Endothelial cells | Cell Marque/Roche Ventana CD31 (JC70) | Ready-to-use / Mouse mAb (JC70) |
| LYVE1 | Liver sinusoidal endothelial cells | Abcam ab219556 | 1:5000 / Rabbit mAb (EPR21857) |

## **Table S1. Primary antibodies used for immunohistochemistry (IHC) of human liver slices.**

## **Table S2: Donor demographics, surgical indication, sample allocation across experiments (Parameter test, Viability evaluation, Drug treatment, IHC, Functionality, RNA-seq), and donor-level viability scores under all tested culture conditions (n = 36 donors, P1–P36).**

|  | **Forward (5'-3')** | **Reverse (5'-3')** |
| --- | --- | --- |
| **GAPDH** | AGGTGAAGGTCGGAGTCAACG | TGGAAGATGGTGATGGGATTT |
| **HMGCR** | TGATTGACCTTTCCAGAGCAAG | CTAAAATTGCCATTCCACGAGC |
| **ALAS1** | CGCCGCTGCCCATTCTTAT | TCTGTTGGACCTTGGCCTTAG |
| **FASN** | AAGGACCTGTCTAGGTTTGATGC | TGGCTTCATAGGTGACTTCCA |
| **FZD4** | CCTCGGCTACAACGTGACC | TGCACATTGGCACATAAACAGA |

## **Table S3: List of primer sequences.**

## **Table S4: Differential expression results for all genes (DESeq2)**

# **Supplementary Figures**

## **Figure S1: *Ex vivo* high-oxygen culture setup for human precision-cut liver slices (hPCLS).**

Culture plates containing hPCLS are placed on an orbital shaker inside a sealed acrylic oxygen chamber positioned within a standard CO₂ incubator. The chamber is supplied with a humidified high-oxygen gas mixture (≈85% O₂, 5% CO₂, balance N₂), while gentle orbital shaking promotes medium exchange at the air–liquid interface.

## **Figure S2: Supportive readouts for viability assessment, oxygen exposure and tissue baseline characteristics.**

**(A)** Comparison of H&E-based viability scoring and MTS metabolic viability readouts in matched hPCLS samples, n = 9 matched samples from three independent donors. Lines connect paired H&E and MTS values from the same sample. Correlation was assessed using Spearman rank correlation (ρ = 0.93, two-sided P = 0.00024). **(B)** Dissolved oxygen measurements in culture medium under standard incubator conditions (21% O₂) and in the oxygen chamber (85% O₂), n = 12 samples from seven independent donors. Individual traces represent repeated medium oxygen measurements. **(C)** Wet tissue weight per slice and **(D)** protein content per mg tissue at D0, D3 and D5 under control and optimised hPCLS-EV conditions, n = 11 independent donors . D0 represents the pre-culture baseline; D3 and D5 represent post-initiation culture time points. Dots represent individual slice-level measurements, and horizontal lines indicate mean values.

## **Figure S3: Preservation of cellular microenvironment complexity under optimised culture conditions.**

Liver tissue slices from seven independent donors were analysed at D0, D3 and D5 by immunohistochemical staining for HepPar-1, Ki-67, CD68, CD56 and CD11c. D0 represents the pre-culture baseline; D3 and D5 represent post-initiation culture time points. (**A**) Representative images of stained sections at each time point. Scale bars, 100 µm. (**B–F**) Quantitative analysis of marker-positive cell populations across culture time points, n = 7 independent donors. Each dot represents the mean value from one donor, derived from two donor-matched slices where available. Bars show mean percentages ± s.d. Statistical analysis was performed on donor-level values using the Friedman test with Dunn’s multiple-comparison correction. ns = not significant; *P < 0.05. (**G**) Summary overview of the quantitative results across all assessed markers and donors. Each dot represents the donor-level value for the indicated marker at the indicated time point.
